# Psychometric evaluation of the 21-item Depression, Anxiety and Stress Scale (DASS-21) among Afghans

**DOI:** 10.1186/s12888-025-07613-6

**Published:** 2025-11-20

**Authors:** Ahmad Neyazi, Bilal Ahmad Rahimi, Abdul Qadim Mohammadi, Prakasini Satapathy, Yasamin Shikhulislamy, Farima Qaderi, Bibi Shahd Qarizada, Habibah Afzali, Mehrab Neyazi, Mark D. Griffiths

**Affiliations:** 1 Afghanistan Center for Epidemiological Studies, Herat, Afghanistan; 2 Faculty of Medicine, Kandahar University, Kandahar, Afghanistan; 3 Department of Mental Health, Herat Regional Hospital, Herat, Afghanistan; 4 Center for Global Health Research, Saveetha Institute of Medical and Technical Sciences, Saveetha Medical College and Hospital, Saveetha University, Chennai, India; 5 Afghanistan Medical Students Association, Herat, Afghanistan; 6 Department of Psychology, Nottingham Trent University, 50 Shakespeare Street, Nottingham, NG1 4FQ UK

**Keywords:** DASS-21, Anxiety, Depression, Stress, Psychometric evaluation, Afghanistan

## Abstract

**Background:**

Depression, anxiety, and stress are psychological mood states that have close relationships. Moreover, they are influenced by situational factors such as living in conflict zones (e.g., Afghanistan). The World Health Organization’s assessment suggests that a significant proportion, specifically one-tenth, of the population residing in areas of conflict endure varying degrees of mental health disorders, ranging from moderate to severe. The most used psychometric instrument to assess these mood states is the 21-item Depression, Anxiety and Stress Scale (DASS-21). However, the scale has not been validated in Dari, the most spoken language in Afghanistan.

**Methods:**

A cross-sectional investigation was undertaken between June and September of 2023 across various locales within Afghanistan, aiming to assess the psychometric attributes of the Dari iteration of the DASS-21. The sample comprised 1318 participants (69% females) with mean age of 32 years.

**Results:**

The total scale demonstrated excellent internal consistency (Cronbach’s α = 0.94), with strong reliability for the depression (α = 0.860), anxiety (α = 0.832), and stress (α = 0.883) subscales. Test–retest reliability was high for all subscales (ICC range: 0.784–0.916). Significant positive correlations were observed between DASS-21 subscales and scores on the 20-item Center for Epidemiologic Studies Depression Scale (CES-D-20) and the 28-item General Health Questionnaire (GHQ-28), supporting convergent validity.

**Conclusions:**

The findings provide strong evidence that the Dari version of the DASS-21 is a psychometrically sound instrument for assessing depression, anxiety, and stress among Dari-speaking Afghan adults. The scale demonstrated excellent internal consistency, good test–retest reliability, and strong convergent validity, supporting its use in research and public health contexts for screening psychological distress. However, given the non-clinical and predominantly female sample, further studies in clinical populations and more diverse sociocultural groups are recommended to confirm its generalizability and diagnostic utility.

**Supplementary Information:**

The online version contains supplementary material available at 10.1186/s12888-025-07613-6.

## Introduction

Globally, a billion individuals are afflicted by mental, neurological, and substance use disorders [1]. According to the World Health Organization (WHO), in 2019, depression and anxiety were the second and sixth leading causes of global years lived with disability (YLD), contributing 5.6% and 3.4% of the total YLDs, respectively [2]. YLD is a metric that combines both the prevalence of a disease and its severity, measuring the number of years lived with a disability, whether physical or mental. In this case, the 5.6% and 3.4% figures reflect the global burden of mental health disorders (specifically depression and anxiety) relative to all other diseases and conditions contributing to disability worldwide. According to a recent large-scale cross-sectional study published in 2024, the prevalence rates of depression, anxiety, and stress in Afghanistan (where the present study was conducted) were found to be 72.05%, 71.94%, and 66.49%, respectively [3]. Depression, anxiety, and stress are psychological states that are closely related, yet distinct. While depression and anxiety are both mood states characterized by persistent emotional and cognitive disturbances, stress is typically understood as a response to external pressures or challenges. Chronic stress can contribute to the development of mood disorders such as depression and anxiety. In the 21-item Depression, Anxiety and Stress Scale (DASS-21), stress is assessed as a dimension of psychological distress that reflects negative emotional states such as irritability and tension, rather than a sustained mood state (e.g., depression or anxiety). This distinction is crucial for understanding how the DASS-21 assesses these factors separately, because each represents different aspects of psychological distress [4]. Chronic psychological stressors might lead to the development of depression or anxiety disorders or both [5]. The DASS can distinguish between everyday stress and clinical disorders such as depression and anxiety. Its tripartite model is designed to assess these three conditions as separate but related entities [6].

Internationally, research has investigated the psychometric characteristics of the 21-item Depression, Anxiety, Stress Scale across diverse demographic groups, encompassing general adults [7], adolescents [8, 9], college students [10, 11], children under 14 years of age [12], schoolchildren [13], hospital patients [14, 15], and individuals within specific professions such as hospital staff [16].

Psychological mood states are influenced by situational factors such as living in conflict zones (e.g., Afghanistan). According to the World Health Organization (2019), it is estimated that one-tenth of individuals residing in conflict-affected regions endure moderate to severe mental health disorders [17]. Due to conflicts and wars for the last four decades, the mental health of Afghans has been negatively impacted. In a study conducted in Mazar-i-Sharif, located in northern Afghanistan, in 2022, it was found that girls exhibited elevated levels of anxiety and depression when compared to boys [18]. In a study conducted in 2022, investigating mental health and suicidality among university students in Afghanistan, findings showed that 69.7% exhibited clinical indicators of depression subsequent to the Taliban takeover in 2021 [19]. According to a 2002 study by the Centers for Disease Control and Prevention (CDC), the prevalence of depression was 73% among Afghan women [20]. Similarly, recent studies reported that the prevalence of depression among Afghan women conducted before and after the government fell to the Taliban was 79.1% and 80.4%, respectively [21, 22].

Historically, Afghanistan’s psychological well-being has been deeply affected by decades of conflict, starting with the Soviet invasion in the 1980s, followed by civil war and the Taliban’s rise in the 1990s. After the U.S. invasion in 2001, the country experienced two decades of foreign military presence and continued instability. The Taliban’s return to power in 2021 worsened existing mental health issues due to societal changes and renewed violence. However, Afghanistan’s mental health crisis is a result of these prolonged conflicts, including the trauma from U.S. military operations and the breakdown of infrastructure. Gender also plays a critical role, with studies showing that girls are more affected by anxiety and depression due to gendered exposure to violence and cultural pressures [23].

A national depression and anxiety survey in Afghanistan showed significant exposure of Afghans to traumatic occurrences, with 64.7% reporting personal experiences of at least one traumatic life event [24]. Gender (i.e., being female), poor overall health status, poor nutritional condition, clinical presentation of COVID-19, and concurrent comorbidities (excluding diabetes and hypertension) had significant associations with mental health disorders [25].

A study conducted in Kabul (Afghanistan) investigated the correlation between food insecurity and prevalent mental health issues among a cohort of 421 women of reproductive age [26]. They used the DASS-21 to assess major mental health problems. The authors reported a prevalence of 89.4% for depression, 90.8% for anxiety, and 85.7% for stress. There were statistically significant associations between food insecurity and all three mood states (i.e., depression, anxiety, and stress). Mental health challenges in Afghanistan are worsened by food insecurity, which has been intensified by ongoing conflict and the COVID-19 pandemic. Conflict disrupts food production and access, exacerbating poverty and stress, which are associated with higher rates of depression, anxiety, and stress. The COVID-19 pandemic further strained mental health by increasing fear, uncertainty, and isolation, particularly within an already overburdened healthcare system. These factors, combined with the enduring impact of conflict, have significantly worsened the psychological distress experienced by the population [26, 27]. Although the DASS-21 has been used in Afghan studies previously, no previous study has ever examined its psychometric properties. Therefore, the main objective of the present study was to evaluate the psychometric properties of the DASS-21 among Afghans.

## Materials and methods

### Participants and procedure

The present study used a convenience sample comprising 1318 adults (409 males and 909 females) from four provinces of Afghanistan (i.e., Herat, Badghis, Samangan, and Mazar-e-Sharif). The data were collected between June and September 2023 with the help of skilled data collectors. Participants completed a self-administered survey including sociodemographic details and the DASS-21.

A cohort comprising 2500 residents situated within the specified four provinces was directly solicited for participation in the study through in-person engagement conducted outdoors in areas proximate to their residences or places of employment. Of this group, 1318 individuals satisfied the predetermined inclusion criteria and consented to participate in the study (response rate: 52.7%). In order to qualify for participation in the study, prospective participants were required to fulfill specific predefined criteria. More specifically, they needed to meet the following conditions: (i) be permanent inhabitants of Afghanistan, (ii) be aged 18 years or older, (iii) possess proficiency in comprehending and utilizing the Dari language, and (iv) demonstrate capability to provide written informed consent.

The 1318 participants were aged between 18 and 100 years (mean age = 32.0 years [SD ± 14.0]). Over two-thirds of the participants were female (69%). Over one-third of the participants reported being single (36.3%). More than two-thirds of the participants resided in urban areas (67.1%). Nearly four-fifths of the participants reported that they had a low-income economic status (79.7%). Approximately two-thirds of the participants experienced a traumatic event during the past month (65.3%) (Table 1).

**Table 1:** Characteristics of participants (*N* = 1318)

Characteristic	
Age (mean ± SD)	31.99 yrs (± 14.05)
Age group, n (%)	
18–20 years	341 (25.9)
21–25 years	239 (18.1)
26–30 years	208 (15.8)
31–40 years	224 (17.0)
41–50 years	168 (12.7)
> 50 years	138 (10.5)
Gender, n (%)	
Male	409 (31.0)
Female	909 (69.0)
Marital status, n (%)	
Single	478 (36.3)
Married	840 (63.7)
Residency, n (%)	
Urban	884 (67.1)
Rural	434 (32.9)
Economic status, n (%)	
High income	54 (4.1)
Middle income	213 (16.2)
Low income	1051 (79.7)
Experienced traumatic event in past month, n (%)	
Yes	860 (65.3)
No	458 (34.7)

### Measures

#### Socio-demographics

Demographic questions were included to gather essential participant information, including age, gender, marital status, residency, economic status, and experience of traumatic events within the past month.

#### Depression, Anxiety and Stress Scale (DASS-21)

The DASS-21 (Lovibond & Lovibond, 1995) was used to assess depression, anxiety, and stress levels. The scale comprises 21 items (e.g., *“I felt I was close to panic”*), rated on a four-point scale from 0 (“*never”*) to 3 (“*most or all of time”*). Scores are calculated individually for each subscale ranging from 0 to 21 (seven items each), with an aggregate score ranging from 0 to 63. Higher cumulative scores on the scale signify heightened severity in depression, anxiety, or stress. The psychometric properties of the scale are reported in the ‘Results’ section. The translated scale can be found in the Supplementary Materials.

***General Health Questionnaire-28 (GHQ-28)***: The GHQ-28 [28, 29] assesses psychological well-being across four dimensions (i.e., *“somatic symptoms*,* anxiety and insomnia*,* social dysfunction*,* and severe depression”*), and was used to test convergent validity with the Dari DASS-21. The scale comprises 28 items (e.g., *“Felt that life is entirely hopeless”*), rated on a four-point scale from 0 (“*better than usual”*) to 3 (“*much worse than usual”*). Scores are calculated individually for each subscale ranging from 0 to 21 (seven items each), with an aggregate score ranging from 0 to 84. Higher cumulative scores on the scale signify heightened somatic symptoms, anxiety and insomnia, social dysfunction, and severe depression. There is no Dari version of the GHQ-28 so the Persian version was adapted for use in the present study. The Cronbach’s alpha in the present study for the total scale was excellent (0.936). In the present study, the Cronbach alphas for the subscales were all very good: somatic symptoms (0.890), anxiety and insomnia (0.841), social dysfunction (0.812), and severe depression (0.887).

#### Center for Epidemiologic Studies Depression Scale (CES-D-20)

The CES-D-20 [30, 31] assesses the presence and severity of depressive symptoms, and was used to test convergent validity with the Dari DASS-21. The scale comprises 20 items (e.g., *“I did not feel like eating; my appetite was poor”*), rated on a four-point scale from 0 (“*rarely or none of the time”*) to 3 (“*most or all of the time”*). Scores on the scale range from 0 to 60, with higher scores denoting heightened depressive symptoms. In the present study, the Cronbach’s alpha was very good (0.888).

### Translation process

Previous studies that used the DASS-21 in Afghanistan did not describe their translation process or publish the Dari version they used. Therefore, a meticulous and comprehensive translation of the DASS-21 from English to Dari was conducted in line with the guidelines of the American Educational Research Association (AERA), American Psychological Association (APA), and National Council on Measurement in Education (NCME) Standards for Educational and Psychological Testing [32]. Experienced translators (native Dari speakers), were chosen to independently translate the DASS-21 items and instructions from English to Dari, with a focus on clarity and cultural appropriateness, while documenting their translation decisions and any encountered challenges. These translations underwent thorough scrutiny by a four-person bilingual expert panel comprising psychologists and linguists to address any disparities and ensure the preservation of the intended meanings. Additionally, a separate bilingual translator performed a back-translation of the Dari version into English to pinpoint any discrepancies or mistranslations, which were meticulously reviewed and rectified by the expert panel. Cognitive interviews with Dari-speaking individuals were conducted to assess the comprehensibility, clarity, and cultural relevance of the translated items, prompting revisions as necessary.

### Data analysis

Internal consistency was assessed using Cronbach’s alpha, with values exceeding 0.70 considered satisfactory. Test-retest reliability was evaluated using the Interclass Correlation Coefficient, and a value of 0.70 or above considered satisfactory. The results depict the ICC values with their corresponding 95% confidence intervals (CI), F-test statistics, and associated *p*-values for the total scale and subscales.

Pearson correlation analyses were employed to assess convergent validity, comparing DASS-21 scores (depression, anxiety, and stress subscales) with the GHQ-28 (depression and anxiety subscales), and CES-D 20 (total score and subscale scores). The correlation coefficients, along with their respective *p*-values, provide the degree of association between these psychological measures. Statistical analyses were conducted utilizing IBM SPSS Statistics software, version 26, designed for the Windows operating system.

## Results

Table 2 presents the item response frequency for each item along with classical test theory (CTT) statistics. A quarter of the participants (26.0%) reported that they occasionally found it *“hard to wind down”.* Nearly one-quarter of the participants (24.6%) reported sometimes of *“experiencing breathing difficulty”* in the past week. Approximately one-fifth of the participants (21.0%) indicated most or all of the time *“experiencing trembling”* in the past week. Item 19, in the anxiety sub-scale (*“I was aware of the action of my heart in the absence of physical exertion*”) was the least frequently endorsed response in the *“most or all of the time”* category, with only 11.8% of the total participants selecting this option. Overall, the Cronbach’s alpha for the total DASS-21 was 0.94, and for depression, anxiety and stress subscales, the alpha values were 0.860, 0.832, 0.883 respectively, which indicate very good internal consistency (Table 2).

**Table 2:** Abbreviated item content, response category percentages, and classical test theory statistics of DASS-21 items

No	Item Description	Never (0)	Sometimes (1)	A lot of the time (2)	Most or all of time (3)	Corrected Item–total correlation	Cronbach’s alpha if the item is dropped
*Anxiety subscale*		%					
2	I was aware of dryness of my mouth.	28.1	25.9	19.4	26.6	0.672	0.936
4	I experienced breathing difficulty.	46.1	24.6	14.5	14.8	0.530	0.939
7	I experienced trembling (e.g., in the hands).	32.5	24.1	22.4	21.0	0.654	0.937
9	I was worried about situations in which I might panic and make a fool of myself.	40.4	26.6	18.3	14.7	0.593	0.938
15	I felt I was close to panic.	37.3	27.5	18.9	16.2	0.646	0.937
19	I was aware of the action of my heart in the absence of physical exertion.	44.8	23.8	19.6	11.8	0.419	0.940
20	I feel scared without any good reason.	46.1	22.5	19.0	12.5	0.520	0.939
*Depression subscale*							
3	I couldn’t seem to experience any positive feeling at all.	35.7	26.7	21.1	16.5	0.673	0.936
5	I find it difficult to work up the initiative to do things.	30.0	23.2	23.0	23.7	0.638	0.937
10	I felt that I had nothing to look forward to.	32.9	22.1	18.5	26.6	0.682	0.936
13	I felt down-hearted and blue.	26.8	22.8	19.0	31.4	0.752	0.935
16	I was unable to become enthusiastic about anything.	31.9	26.9	25.7	15.5	0.638	0.937
17	I felt I wasn’t worth much as a person.	48.4	22.9	18.9	9.8	0.563	0.938
21	I feel that life is meaningless.	38.6	15.8	16.2	29.4	0.623	0.937
*Stress subscale*							
1	I find it hard to wind down.	23.3	26.0	21.0	29.7	0.695	0.936
6	I tend to over-react to situations.	23.9	21.3	24.3	30.5	0.551	0.938
8	I feel that I was using a lot of nervous energy.	22.2	22.8	28.5	26.5	0.523	0.939
11	I find myself getting agitated.	24.4	20.6	20.1	34.9	0.774	0.935
12	I find it is difficult to relax.	23.3	24.8	19.1	32.8	0.786	0.934
14	I was intolerant of anything that kept me from getting on with what I was doing.	25.9	25.5	17.2	31.4	0.649	0.937
18	I feel that I was rather touchy.	19.0	20.7	21.6	38.7	0.680	0.936

The findings showed a robust and consistent level of test-retest reliability across all dimensions. More specifically, the anxiety subscale had an ICC of 0.916 (95% CI: 0.886–0.937), reflecting a high degree of agreement between the two test administrations. Similarly, the depression and stress subscales exhibited ICC values of 0.870 (95% CI: 0.824–0.903) and 0.784 (95% CI: 0.722–0.848), respectively, affirming good reliability of these constructs over time. The overall total score of the DASS-21 had an ICC of 0.924 (95% CI: 0.898–0.944), indicating a reliable measure encompassing all subscales (Table 3).

**Table 3:** The interclass correlation coefficient between two rounds of the DASS-21 (*n* = 172)

Scale/Subscale	ICC^a^ (95% CI^b^)	*p*-value
Anxiety	0.916 (0.886–0.937)	**< 0.001**
Depression	0.870 (0.824–0.903)	**< 0.001**
Stress	0.784 (0.722–0.848)	**< 0.001**
Total	0.924 (0.898–0.944)	**< 0.001**

^a^ ICC = Intra-class correlation coefficient. Two-way random

^b^ CI = Confidence interval

**Table 4:** Pearson correlation coefficients between DASS-21 depression subscale, DASS-21 anxiety subscale, DASS-21 stress subscale, CES-D-20, GHQ-28 anxiety subscale, and GHQ-28 depression subscale

Variables	1	2	3	4	5	6	7	8
1. DASS-21 depression subscale	1	-	-	-	-	-	-	-
2. CES-D-20	0.754* **< 0.001**	1	-	-	-	-	-	-
3. GHQ-28 depression subscale	0.710* **< 0.001**	0.574* **< 0.001**	1	-	-	-		
4. DASS-21 anxiety subscale	0.755* **< 0.001**	0.720* **< 0.001**	0.597* **< 0.001**	1	-	-		
5. GHQ-28 anxiety subscale	0.777* **< 0.001**	0.685* **< 0.001**	0.648* **< 0.001**	0.764* **< 0.001**	1	-		
6. DASS-21 stress subscale	0.817* **< 0.001**	0.771* **< 0.001**	0.617* **< 0.001**	0.685* **< 0.001**	0.739* **< 0.001**	1		
7. DASS-21 total score	0.940* **< 0.001**	0.814* **< 0.001**	0.698* **< 0.001**	0.881* **< 0.001**	0.825* **< 0.001**	0.919* **< 0.001**	1	
8. GHQ total score	0.824* **< 0.001**	0.718* **< 0.001**	0.840* **< 0.001**	0.756* **< 0.001**	0.878* **< 0.001**	0.767* **< 0.001**	0.850* **< 0.001**	1

*Correlation is significant at 0.01 level

The results indicated substantial and statistically significant positive correlations between the DASS-21-depression subscale scores and scores on both the CES-D-20 (*r* = 0.754, *p* < 0.001) and GHQ-28 depression subscale (*r* = 0.710, *p* < 0.001). Similarly, scores on the DASS-21-anxiety subscale indicated strong positive correlations with scores on both the CES-D-20 (*r* = 0.755, *p* < 0.001) and GHQ-28 anxiety subscale (*r* = 0.777, *p* < 0.001). Scores on the DASS-21 stress subscale indicated comparable positive associations with scores on the CES-D-20 (*r* = 0.817, *p* < 0.001) and GHQ-28 anxiety subscale (*r* = 0.739, *p* < 0.001). There were also significant positive correlations between total scores on the DASS-21 and total scores on both the CES-D-20 (*r* = 0.814, *p* < 0.001) and GHQ-28 (*r* = 0.850, *p* < 0.001) (Table 4).

## Discussion

The present study evaluated the psychometric properties of the Dari version of the DASS-21 among Afghans. To date, the DASS-21 and DASS-42 have been translated into 57 different national languages, including Dari, Pashto, Persian, Arabic, Urdu, Hindi, Indonesian, Malaysian, Chinese, Thai, and Turkish [33]. Due to the fact that studies have been carried out in many different cultures, variations regarding the instrument’s internal structure and psychometric properties have been observed.

Various models have been observed in the literature, encompassing a range of structures such as a three-factor model, a second-order model comprising three factors (anxiety, depression, and stress), a tripartite model (consisting of anhedonia, physiological hyperarousal, and a general negative factor), a bifactor model (where items primarily load on a general factor of distress alongside specific factors of stress, depression, and anxiety), a two-factor model (comprising depression and anxiety/stress), a four-factor model (including a general factor of distress, alongside depression, anxiety, and stress factors), and a single-factor model of distress [5, 12, 34]. Previous studies have generally supported the three-factor model (anxiety, depression, and stress) of the DASS-21. Although the present study did not examine the latent structure, the reliability and validity evidence obtained was consistent with findings from these earlier studies [5].

The Cronbach’s alpha for the total DASS-21 was 0.94, and for depression, anxiety and stress subscales, the alpha values were 0.860, 0.832, 0.883 respectively. These findings indicated that the Dari DASS-21 demonstrates very good internal consistency. The findings also indicated a robust and consistent level of test-retest reliability for all subscales, with an ICC of 0.916, 0.870, and 0.784 for anxiety, depression, and stress, respectively. The ICC for the total DASS-21 was 0.924. These findings demonstrate that the Dari version of DASS-21 is a reliable psychometric instrument with excellent internal consistency. In the original validation study, the Cronbach’s alpha coefficients were 0.91 for depression, 0.81 for anxiety, and 0.89 for stress [4]. The findings in the present study concur with those of the original study.

The overall Cronbach alpha concurs with (and is even better in some instances) than other studies from India (0.85 among adults aged between 18 and 25 years) [35], Bangladesh (0.98 among university students) [36], China (0.89 among school teachers) [37], Turkey (0.82 among non-clinical population) [38], Vietnam (0.761 among adults) [39], Malaysia (0.87 among students, teachers, and non-teaching staff of a university) [40], Indonesia (0.91 among the general population) [41], and Greece (0.97 among the adult population) [42].

In the present study, and as expected, significant positive associations were found between total score on the DASS-21 and total scores on both the CES-D-20 and the GHQ-28. There were also significant positive associations between the DASS-21-depression subscale scores and scores on both the CES-D-20 and GHQ-28 depression subscales. Similarly, scores on the DASS-21-anxiety subscale were strongly positively associated with scores on both the CES-D-20 and GHQ-28 anxiety subscale. Finally, scores on the DASS-21 stress subscale had positive associations with CES-D-20 and the GHQ-28 anxiety subscale. Combined, all of these findings demonstrate robust convergent validity of the DASS-21 with other scales and subscales assessing the same conditions.

The findings also concur with a 2017 Afghan study conducted with 1310 non-clinical randomly selected participants in Herat city using the DASS-42. In that study, the Cronbach’s alpha values were 0.888 for depression, 0.866 for stress, and 0.833 for anxiety [43]. The marginal discrepancies in outcomes could potentially be attributed to the research methodology, which entailed extended time intervals between the initial and subsequent administrations of the DASS-21, and/or the utilization of varied iterations of the DASS [44].

Previous studies conducted among clinical and non-clinical Canadian adults [45], non-clinical UK adults [46], depressed Canadian adults [47], non-clinical US adults [48], and non-clinical Iranian adults [49] confirmed a three-factor model for DASS-21. However, test-retest reliability of the DASS-21 has not been observed in studies conducted in Australia [50, 51], Italy [52], and China [53]. This occurrence could potentially be attributed to a methodological issue arising from prolonged intervals between the initial and subsequent administrations of the DASS-21 within the research protocol [27].

Despite the strengths of the present study, including the use of well-established psychometric instruments and a relatively large sample, several limitations should be acknowledged. Data were obtained from a non-clinical convenience sample, which may not fully represent individuals with clinically diagnosed depressive, anxiety, or stress disorders. Replication of the present findings among clinical populations is recommended to confirm the diagnostic utility of the Dari DASS-21. The sample was disproportionately female (approximately 69%), which may have introduced gender-related bias and limits the generalizability of the results to the broader Afghan population. This imbalance was primarily attributable to sociocultural and security constraints that restricted data collection from men. The study focused exclusively on Dari-speaking participants from selected provinces, which may reduce the generalizability of the findings to other regions and to Pashto-speaking populations. Future research should consider culturally and linguistically diverse samples to ensure broader applicability of the scale. Information regarding participants’ educational attainment was not collected, precluding examination of potential differences in comprehension or interpretation of the DASS-21 items across literacy levels. Reliance on self-report measures may have introduced recall bias and social desirability effects, potentially influencing the accuracy of symptom reporting. Finally, the cross-sectional design precludes conclusions regarding temporal stability or causal relationships. Longitudinal investigations are warranted to assess the stability of the scale scores over time and to further establish the psychometric robustness of the Dari DASS-21 in different populations.

## Conclusion

The findings of the present study provide robust evidence that the Dari version of the DASS-21 is a psychometrically sound instrument for assessing depression, anxiety, and stress among Dari-speaking adults in Afghanistan. The scale demonstrated excellent internal consistency, good test–retest reliability, and strong convergent validity with established measures. These results suggest that the Dari DASS-21 is suitable for use in both research and public health contexts to assess and monitor psychological distress within the Afghan population. Nevertheless, given the non-clinical and predominantly female nature of the sample, as well as the geographic and linguistic limitations, further studies are recommended to validate the instrument among clinical populations, men, and more diverse sociocultural groups. Such investigations will help confirm the generalizability and diagnostic utility of the Dari DASS-21 across different Afghan contexts.

## Supplementary Information

Below is the link to the electronic supplementary material.

Supplementary Material 1

## Data Availability

The datasets utilized and/or analyzed in the course of the present study are accessible from the corresponding author upon reasonable request.
