# Supplementary material for: Psychometric evaluation of the 21-item Depression, Anxiety and Stress Scale (DASS-21) among Afghans
Source: BMC Psychiatry. 2025 Nov 20;25:1186. doi: 10.1186/s12888-025-07613-6 (PMC12751860; doi:10.1186/s12888-025-07613-6)
Supplement: Supplementary file 1 — Supplementary Material 1 [file 12888_2025_7613_MOESM1_ESM.docx]

DASS-21 items

| No | Item | Never (0) | Sometimes (1) | A lot of times (2) | Most or all of time (3) |
| --- | --- | --- | --- | --- | --- |
| 1 | I find it hard to wind down. |  |  |  |  |
| 2 | I was aware of dryness of my mouth. |  |  |  |  |
| 3 | I couldn’t seem to experience any positive feeling at all. |  |  |  |  |
| 4 | I experienced breathing difficulty. |  |  |  |  |
| 5 | I find it difficult to work up the initiative to do things. |  |  |  |  |
| 6 | I tend to over-react to situations. |  |  |  |  |
| 7 | I experienced trembling (e.g., in the hands). |  |  |  |  |
| 8 | I feel that I was using a lot of nervous energy. |  |  |  |  |
| 9 | I was worried about situations in which I might panic and make a fool of myself. |  |  |  |  |
| 10 | I felt that I had nothing to look forward to. |  |  |  |  |
| 11 | I find myself getting agitated. |  |  |  |  |
| 12 | I find it is difficult to relax. |  |  |  |  |
| 13 | I felt down-hearted and blue. |  |  |  |  |
| 14 | I was intolerant of anything that kept me from getting on with what I was doing. |  |  |  |  |
| 15 | I felt I was close to panic. |  |  |  |  |
| 16 | I was unable to become enthusiastic about anything. |  |  |  |  |
| 17 | I felt I wasn’t worth much as a person. |  |  |  |  |
| 18 | I feel that I was rather touchy. |  |  |  |  |
| 19 | I was aware of the action of my heart in the absence of physical exertion. |  |  |  |  |
| 20 | I feel scared without any good reason. |  |  |  |  |
| 21 | I feel that life is meaningless. |  |  |  |  |
